# Glucose variability for cardiovascular risk factors in type 2 diabetes: a meta-analysis

**DOI:** 10.1186/s40200-017-0323-5

**Published:** 2017-11-14

**Authors:** Shuang Liang, Hang Yin, Chunxiang Wei, Linjun Xie, Hua He, Xiaoquan Liu

**Affiliations:** Department of Center of Drug Metabolism and Pharmacokinetics, China Pharmaceutical University, Nanjing, China

## Abstract

**Aims:**

It is consensus that glucose variability (GV) plays an important role in maccomplications of type 2 diabetes, but whether GV has a causal role is not yet clear for cardiovascular disease (CVD). This study sought to explore the effect on GV for CVD risk factors with type 2 diabetes.

**Methods:**

The systematic literature search was performed to identify all GV and CVD risk factors, including total cholesterol (TC), LDL cholesterol (LDL-C), triglyceride (TG), HDL cholesterol (HDL-C), Body Mass Index (BMI), waist circumference (WC), High-Sensitivity C-reactive protein (Hs-CRP), Homeostasis model assessment (HOMA) and carotid intima-media thickness (IMT). Preferred Reporting Items was synthesized for Systematic reviews and Meta Analyses guideline. And the pooled analyses were undertaken using Review Manager 5.3.

**Results:**

Twenty two studies were included with a total of 1143 patients in high glucose variability group (HGVG) and 1275 patients low glucose variability group (LGVG). Among these selected CVD risk factors, HOMA-IR and reduced IMT were affected by GV. HOMA-IR level was significantly lower in LGVG than in HGVG (MD = 0.58, 95% CI: 0.26 to 0.91, *P* = 0.0004), with evidence of heterogeneity between studies (I^2^ = 0%; *P* = 0.47).

Reduced IMT level was significantly lower in LGVG than in HGVG (SMD = 0.28, 95% CI: 0.09 to 0.47, *P* = 0.003), with evidence of heterogeneity between studies (I^2^ = 0%; *P* = 0.48). However, the others were no significant statistical difference.

**Conclusions:**

Among these selected CVD risk factors in type 2 diabetes, minimizing GV could improve insulin resistance and reduced IMT, consistent with a lowering in risk of CVD.

**Electronic supplementary material:**

The online version of this article (10.1186/s40200-017-0323-5) contains supplementary material, which is available to authorized users.

## Introduction

Cardiovascular diseases (CVD) are the major causes of morbidity and mortality in type 2 diabetes [1], which death rate accounts for 75% [2]. It is widely accepted that lipid metabolism, Body Mass Index (BMI), waist circumference (WC), Homeostasis model assessment (HOMA), High-Sensitivity C-reactive protein (Hs-CRP) and carotid intima-media thickness (IMT) are dominant risk factors of cardiovascular disease (CVD) [3–5]. If not adequately controlled, these risk factors would increase CVD events, and they are also significant for clinical.

In prospective epidemiologic studies, the incidence of microvascular complications is directly linked with the degree of hyperglycemia, represented by the glycosylated hemoglobin level(HbA1c), which is expressed as mean blood glucose level during the previous 2 to 3 months [6]. Meanwhile, UKPDS shows that an increase of 1% in HbA1c is associated with an increase of 37% in the risk of retinopathy or kidney disease [7]. However, ACCORD and ADVANCE have failed to provide an additional benefit in CVD with long-standing diabetes [8–10], even after maintaining near-normal HbA1c level in persons with type 2 diabetes. As a corollary, the uncertainty around HbA1c results related to clinical outcomes was augmented. Meanwhile, these findings suggest that near-normal HbA1c does not possibly improve CVD outcomes, so it’s incompleteness need to fill out. Glucose variability(GV) mainly refer to as time in range and is unacceptable in hypo- and hyperglycemic range (<70 and 180 mg/dL, respectively) [11], it has emerged as a key unmet need.

Although GV is emerging as an important dynamic parameter of diabetes control, its clinical importance is not fully characterized. Growing studies have reignited the emphasis that GV is a risk factor for diabetic complication. In general, much studies of GV in vitro laboratory evidence show that GV could increase production of reactive oxygen species and has a detrimental effect on endothelial dysfunction, even CVD [12]. While similar findings have also been shown in clinical studies [13], others have been unable to confirm any association [14]. Understanding mechanism of GV to CVD may help unravel some of the mystery about mac-complication in type 2 diabetes. So we aimed to describe association between CVD risk factor levels and GV in type 2 diabetes, providing opportunities for early diagnosis and targets for novel treatments.

## Methods

### Literature search

This review was performed by the Preferred Reporting Items for Systematic reviews and Meta Analyses guideline [15]. We searched PubMed, EMBASE, Cochrane Library, Web of science, Wan Fang Data and CNKI from 1970 to October 12, 2016 by using text words (diabetes [Title/Abstract]) AND (randomized clinical trial) AND (glucose variability OR glycemic variability OR glucose fluctuation OR glucose instability OR glycemic fluctuation). All relevant abstracts were obtained from our search. References from these studies were reviewed for additional citations and all potential articles.

### Trial eligibility and selection

We included Chinese and English-language, full paper, randomized controlled clinical trials (RCTs) conducted in adult over 18 years of age patients with type 2 diabetes. The search strategy mainly focus on the association between GV and major CVD risk factors with type 2 diabetes, which include BMI [4], WC, TC, TG, HDL, LDL [3], Hs-CRP [5], HOMA and IMT. Studies that captured at least two group of glucose variability, including Mean blood glucose (MBG), coefficient of variation (CV), standard deviation(SD), mean amplitude of glycemic excursions (MAGE), mean of daily differences (MODD), continuous overall net glycemic action(CONGA), standard deviation-glycosylated hemoglobin (SD-HbA1c), and standard deviation-Fasting plasma glucose (SD-FPG) [12, 16], assessed using either self-monitoring of blood glucose (SMBG) or continuous glucose monitoring (CGM) or reported a measure of GV were included in the review, and that were excluded if they had an impaired peripheral arterial disease, renal, liver, coronary heart disease, and stroke on the baseline. By contacting the corresponding authors, attempt to acquire studies that did not report the required data on GV and CVD risk factors.

Quality and characteristics of included studies were assessed regarding the methodological characteristics, statistical analysis, characteristics of the outcome by two reviewers. Where there was disagreement over the eligibility of a study, the article was discussion together and a consensus was reached.

### Data analysis and synthesis

Because there are no generally accepted gold standard for assessing GV and little consensus for most accurate assessment of GV [16], and each has its own advantages and disadvantages, and it is no consistent assessment index of GV in all studies, therefore GV level are divided into two groups to ensure comprehensive. Comparable studies in terms of GV levels were pooled for meta-analysis if they were statistically significant between experimental group and control group, we define that low amplitude of GV was low glucose variability group (LGVG) and high amplitude of GV is high glucose variability group (HGVG).

In this meta-analysis, mean difference (MD) and standardized mean difference (SMD) were applied all the meta-analysis. Random effects models were used to consider study variation. I^2^ index is to estimate heterogeneity, namely used to determine whether differences exist between studies [17]. Heterogeneity is low if I2 < 30%, heterogeneity is moderate if I^2^ is 30% – 50%, and heterogeneity is concluded if *P* < 0.10 and I^2^ > 50% [17]. Analyses were undertaken using Review Manager 5.3.

### Bias assessment

Bias, being caused by literature search and data analysis, can lead to under- or over-estimation of the true intervention effects in clinical trials. In this meta-analysis, publication bias was assessed using Egger’s test [18]. Sensitivity analyses were assessed by removing one study at a time on the pooled estimate.

Note: Body Mass Index (BMI), waist circumference (WC), Total Cholesterol (TC), triglyceride (TG), high-density lipoprotein (HDL), low-density lipoprotein (LDL), C-reactive protein (CRP), Homeostasis model assessment (HOMA) and Length of Inner Metatarsal Tubercle (IMT).

## Results

**Fig. 1:**
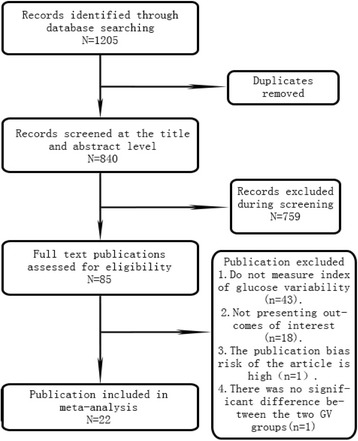
Flow diagram for identifying eligible studies

Among these studies, four authors were contacted for missing data, but no authors provided additional information, so 22 studies are included for full-text review in the meta-analysis, representing a total sample of 1143 patients in HGVG and 1275 patients in LGVG, 1183 studies are removed based on our inclusion and exclusion criteria (Fig. 1), and the vast majority studies were excluded as reviews or not reporting either a measure of GV or no the associated CVD risk factors of interest. These eligible studies contained data on two different groups. And the studies characteristics are summarized in Table 1. Sensitivity analyses revealed that no particular study affected significantly the summary effects for CVD risk factors. Findings from Egger’s test supported the finding that except LDL, others were no publication bias (Table 2). When one article was excluded, the publication bias of LDL is non-existent (P: 0.328).

**Table 1:** Characteristics of included studies

Study,Year	Sample Size (n)(high GV /low GV)	Glucose variability indice	Follow up (month)	Age (mean/arrange)	Men (%)	Ethnics	CVD risk factors
Panwei Mu 2011 [31]	126/124	CV-FBG	3	40	42.8	Xanthous	TC,TG,HDL,LDL,HOMA-IR,HOMA-β
H.J. Yoo 2008 [32]	28/29	MAGE	3	20–80	42.1	Xanthous	TC,TG,HDL,LDL,BMI,WC
Su Guirong 2014 [33]	28/27	MBG SDBG MODD MAGE	12	50	52	Xanthous	TC,TG,HDL,LDL,BMI,HOMA-IR
Shi Dou Lin 2011 [34]	20/20	MBG SD MODD CONGA	6	30–70	57.5	Xanthous	BMI,TC,TG,HDL,LDL
Guoyue Yuan 2015 [35]	104/108	CV MAGE	0.5	49	67.00	Xanthous	BMI,TC,TG,HDL,LDL,Hs-CRP,HOMA-IR
Weiping Sun 2016 [36]	52/51	SD-HbA1c MAGE	6	30–70	52.78	Xanthous	TC,TG,LDL,HDL,HOMA-IR,HOMA-β
HunSung Kim 2013 [37]	17/16	MBG SD MAGE	2	18–80	57.58	Xanthous	TC,TG,HDL,LDL
Claudia De Natale 2009 [38]	13/5	CV MAGE	1	59	66.67	Caucasian	TC,TG,LDL,HDL
Jae-Hyoung Cho 2006 [39]	40/40	SD-HbA1c	30	≥30	61.25	Xanthous	TC,TG,HDL
Yu Qian Bao 2010 [19]	20/20	MBG MODD MAGE	2	34–70	41.3	Xanthous	TC,TG,HDL,LDL,BMI,WC,HOMA-IR,HOMA-β
Helene von Bibra 2016 [40]	48/61	SD-HbA1c MAGE	36	35–85	70.6	Caucasian	TC,TG,HDL,LDL,Hs-CRP,IMT
John B Buse 2016 [41]	159/307	MAGE	13	60.4	57.5	Caucasian	TG,HDL,LDL
Tomoya Mita 2016 [42]	152/151	SD-HbA1c SD-FBG	6	≥30	58.36	Xanthous	IMT,TC, LDL, HDL
Jeannie Tay 2015 [43]	47/46	MBG SD MAGE MODD CONGA-1 CONGA-4	6	35–68	67.74	Caucasian	WC,HOMA-IR,HOMA-β,Hs-CRP,TC,TG,HDL,LDL
Jeannie Tay 2015 [44]	37/41	MAGE SD CONGA-1 CONGA-4	13	35–68	57.39	Caucasian	TC,TG,HDL,LDL,Hs-CRP,HOMA-IR,HOMA-β
Heng Wan 2016 [45]	30/30	SD MBG MAGE	8	30–70	46.5	Xanthous	BMI,TC,TG,HDL,LDL
Huang Zhanqiang 2012 [46]	40/40	CV-FPG SDBG	3	≥60	66.25	Xanthous	TC,TG,HDL,LDL
Qiang Zhou 2008 [47]	56/50	MAGE	6	20–75	62	Xanthous	BMI, WC
Yanzhen Ye 2014 [48]	22/28	MAGE SD	18	46	60.72	Xanthous	TC,TG,HDL,LDL
Ruiting He 2016 [49]	60/60	MBG MAGE SD LAGE	10	56	50	Xanthous	BMI,TC,TG,LDL,HDL
Shuijing Zhou 2012 [20]	23/10	MAGE	24	20–70	50.94	Xanthous	BMI,TG,TC,HDL,LDL,IMT
Wang Ruiping 2015 [50]	29/27	MAGE	1	60–80	58.93	Xanthous	TC,TG,WC

Note: Body Mass Index (BMI), waist circumference (WC), Total Cholesterol (TC), triglyceride (TG), high-density lipoprotein (HDL), low-density lipoprotein (LDL), C-reactive protein (CRP), Homeostasis model assessment (HOMA) and Length of Inner Metatarsal Tubercle (IMT)

**Table 2:** Summary of publication bias with Eggers test

Egger test		t (95%Cl)	*P*
BMI	change	−0.17[4.48,-4.85]	0.88
	final value	0.1[2.61,-2.39]	0.92
WC	final value	1.51[−2.98,8.36]	0.23
TC	change	0.78[4.07,-2.17]	0.47
	final value	−0.5[0.41,-0.66]	0.63
TG	change	0.68[−0.36,0.59]	0.73
	final value	1.27[−0.47,1.85]	0.22
HDL	change	0.16[−3.43,3.90]	0.88
	final value	−0.28[−1.32,1.02]	0.79
LDL	change	0.14[−2.47,2.77]	0.891
	final value	−3.03[−2.81,-0.48]	0.009
HOMA-IR	change	2.85[−1.17,5.74]	0.104
	final value	1.08[−1.11,2.73]	0.329
HOMA-β	change	−2.45[−6.50,4.40]	0.247
	final value	3.87[−0.05,0.92]	0.061
HS-CRP	change	−1.99[−3.72,1.37]	0.185
	final value	−0.76[−4.7,3.28]	0.525
IMT	change	0.71[−17.94,20.08]	0.605

The characteristics of the studied populations varied with baseline values ranging from 42% to 70% for the proportion of males, 18 to 80 years for age. The length of follow-up ranged from 1/2 to 52 months.

### Effect on body mass index and waist circumference

Nine studies are received in BMI, comprising 338 patients of low GV and 353 patients of high GV, and baseline mean values range from 23 (kg/m^2^) to 26 (kg/m^2^). Reduction in BMI was observed in 4 of the 10 studies. The meta-analysis revealed that BMI levels were not significantly higher in HGVG than in LGVG (mean difference [MD] = 0.28 kg/m^2^ (95% confidence interval [95% CI] -0.1 to 0.67) with evidence of heterogeneity between studies (I^2^ = 32%; *P* = 0.16), and reduced BMI levels also did not reach statistical significance (MD = 0.01 kg/m^2^, 95% CI: = − 0.07 to 0.09; I^2^ = 23%; *P* = 0.28) (Table 3, Additional file 1: Figure S1).

**Table 3:** Summary of results for CVD risk factors

CVD risk factors		Population	No.of studies	No.of patients		Test of association			Test of heterogeneity		
				H GV	L GV	MD	95%Cl	*P*-value	Model	*P*-value	I^2^
BMI	change	overall	4	218	225	0.01	[−0.07,0.09]	0.82	R	0.28	23%
	final value	overall	9	353	338	0.28	[−0.10,0.67]	0.15	R	0.16	32%
WC	change	overall	2	84	87	1.35	[−1.13,3.83]	0.29	R	0.81	0%
	final value	overall	5	165	159	1.11	[−0.99,3.22]	0.30	R	0.49	0%
TC	change	overall	7	468	488	−0.12	[−0.26,0.01]	0.07	R	0.18	33%
	final value	overall	16	661	639	−0.03	[−0.06,0.00]	0.06	R	0.78	0%
TG	change	overall	6	317	338	0.19	[0.07,0.30]	0.002	R	0.17	36%
	final value	overall	16	807	939	0.02	[−0.07,0.11]	0.67	R	0.15	28%
HDL	change	overall	16	759	891	0.02	[−0.02,0.05]	0.36	R	0.12	31%
	final value	overall	7	451	474	−0.01	[−0.05,0.03]	0.70	R	0.24	24%
LDL	change	overall	16	779	911	−0.01	[−0.10,0.07]	0.74	R	0.21	21%
	final value	overall	7	468	483	−0.05	[−0.13,0.02]	0.13	R	0.57	0%
HOMA-IR	change	overall	4	247	256	0.18	[−0.00,0.37]	0.05	R	0.43	0%
	final value	overall	7	386	378	0.58	[0.26,0.91]	0.0004	R	0.47	0%
HOMA-β	change	overall	3	147	144	8.44	[−4.53,21.4]	0.2	R	0.5	0%
	final value	overall	4	239	236	1.53	[−2.94,6.00]	0.5	R	0.95	0%
HS-CRP	change	overall	4	255	271	0.33	[−0.09,0.76]	0.12	R	0.33	12%
	final value	overall	4	179	178	−0.24	[−0.73,0.25]	0.33	R	0.60	0%
IMT	change	overall	3	224	226	0.28 (SMD)	[0.09,0.47]	0.003	R	0.48	0%

Five studies comprise 165 patients of high GV and 159 patients of low GV in WC. The meta-analysis revealed that waist circumference level was not associated with glucose variability level (MD = 1.11 cm, 95% CI: = −0.99 to 3.22), with evidence of heterogeneity between studies (I^2^ = 0%; *P* = 0.49) and reduced WC levels also did not reach statistical significance (MD = 1.35, 95% CI: = − 1.13 to 3.83; I^2^ = 0%; *P* = 0.81) (Table 3, Additional file 1: Figure S2).

### Effect on insulin secretion and insulin resistant

**Fig. 2:**
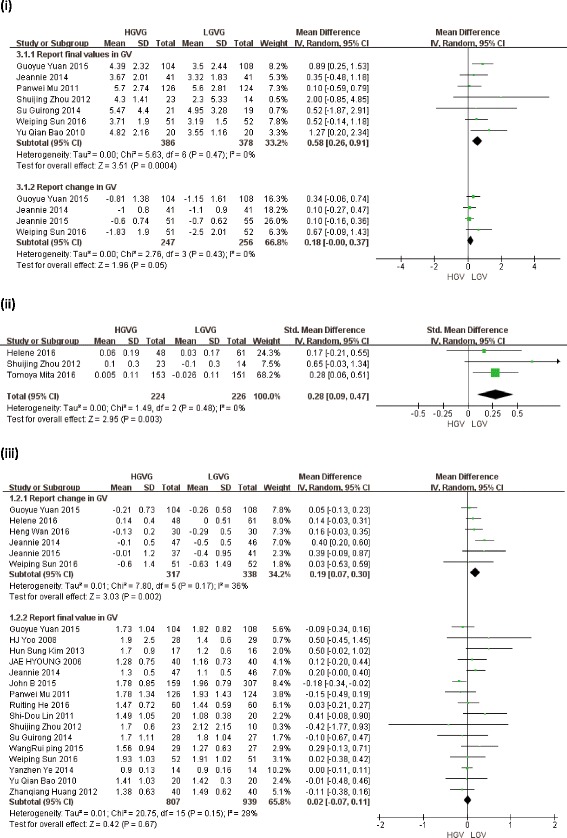
Forest plots of the effect of glucose variability for CVD risk factors in type 2 diabetes patients, showing differences in outcomes of trials with LGVG and HGVG. (i) Effect of GV on HOMA-IR. (ii) Effect of GV on IMT. (iii) Effect of GV on TG. (CL: confidence interval. LGVG: low glucose variability group. HGVG: high glucose variability group. IMT: carotid intima-media thickness TG: triglyceride

Eight studies of HOMA-IR comprise 406 patients of high GV and 399 patients of low GV. Reduction in HOMA-IR was observed in 2 [19, 20] of the 8 studies. The meta-analysis revealed that HOMA-IR level was significantly lower in LGVG than in HGVG (MD = 0.58, 95% CI: = 0.26 to 0.91, *P* = 0.0004), with evidence of heterogeneity between studies (I^2^ = 0%; *P* = 0.47), however, reduced HOMA-IR were not statistical significance (MD = 0.18, 95% CI: = − 0.00 to 0.37; I^2^ = 0%; *P* = 0.43) (Table 3, Fig 2).

Six studies of HOMA-β comprise 302 patients of high GV and 299 patients of low GV. One of five studies inβ-cell function was an increase after lower GV. The pooled weighted mean difference was 1.53 (95% CI = −2.94 to 6.00, *P* = 0.5), with evidence of heterogeneity between studies (I^2^ = 0%; *P* = 0.95). And HOMA-β of the pooled mean change was 8.44 (95% CI = −4.53 to 21.4, *P* = 0.2), with evidence of heterogeneity between studies (I^2^ = 0%; *P* = 0.5) (Table 3, Additional file 1: Figure S7). Whatever final levels and changes of HOMA-β both were no association with GV.

### Effect on lipid metabolism

The meta-analysis revealed that lipid metabolism level was not associated with glucose variability based on TC, TG, HDL and LDL levels. Eighteen studies about TC, it is that MD = −0.03 mmol/l, 95% CI: = −0.06 to 0.00, with evidence of heterogeneity between studies (I^2^ = 0%; *P* = 0.78) (Table 3). Twenty-two studies about TG, it is that MD = 0.02 mmol/l, 95% CI: = −0.07 to 0.11, with evidence of heterogeneity between studies (I^2^ = 28%; *P* = 0.15) (Table 3, Fig 2). Eighteen studies about HDL, it is that MD = −0.01 mmol/l, 95% CI: = −0.05 to 0.03, with evidence of heterogeneity between studies (I^2^ = 24%; *P* = 0.24) (Table 3, Additional file 1: Figure S3). Twenty studies about LDL, it is that MD = −0.05 mmol/l, 95% CI: = −0.13 to 0.02, with evidence of heterogeneity between studies (I^2^ = 0%; *P* = 0.57) (Table 3, Additional file 1: Figure S4).

However, we found that GV were associated with reduced TG (MD = 0.19 mmol/l; 95% CI: [0.07, 0.3]; I^2^ = 36%; *P* = 0.17), and a trend towards reduced others’ levels which did not reach statistical significance: TC (MD = −0.12 mmol/l; 95% CI: [−0.26, 0.01]; I^2^ = 33%; *P* = 0.18), HDL (MD = 0.02 mmol/l; 95% CI: [−0.02, 0.05]; I^2^ = 31%; *P* = 0.12) and LDL (MD = −0.01 mmol/l; 95% CI: [−0.10, 0.07]; I^2^ = 21%; *P* = 0.21) (Table 3).

### Effect on inner metatarsal tubercle and high sensitivity C reactive protein

Seven studies of Hs-CRP comprise 417 patients of high GV and 426 patients of low GV. The meta-analysis revealed that Hs-CRP level was not associated with glucose variability (MD = −0.24 ng/ml, 95% CI: = −0.73 to 0.25, *P* = 0.33), with evidence of heterogeneity between studies (I^2^ = 0%; *P* = 0.6) and reduced WC levels also did not reach statistical significance (MD = 0.33 ng/ml, 95% CI: = − 0.09 to 0.76; I^2^ = 12%; *P* = 0.33) (Table 3, Additional file 1: Figure S6).

Three studies of IMT comprise 224 patients of high GV and 226 patients of low GV. The meta-analysis revealed that reduced IMT level was significantly lower in LGVG than in HGVG (SMD = 0.28 mm, 95% CI: = 0.09 to 0.47, *P* = 0.003), with evidence of heterogeneity between studies (I^2^ = 0%; *P* = 0.48) (Table 3, Fig. 2).

## Discussion

The meta-analysis focuses on how GV affect CVD risk factors among 2 diabetes patients, as L Nalysnyk reported that GV was a significant positive association with the development or progression of diabetic retinopathy, even cardiovascular events and mortality [21]. This meta-analysis showed that glucose variability might affect IMT and insulin resistant. However, the effects of GV on BMI, WC, HOMA-β, lipid metabolism and Hs-CRP were not statistically significant. At the same time, Brohall G reported that impaired glucose tolerance showed a higher IMT [22]. That explained that it might be association among GV, IMT and insulin resistant, in order to provide opportunities for novel treatments.

IMT has been shown a significant predictor of CVD patients [23]. In this meta-analysis, minimizing GV is accompanied by a reduction of IMT with an estimated magnitude between 0.09 and 0.47 mm, which is consistent with an estimated 11% to 59% reduction in risk of myocardial infarction and a 13% to 70% reduction in risk of stroke [24]. Meanwhile, some studies found that IMT was associated with Phosphoinositide 3-kinase (PI3K) [25] and AMPK pathway [26], suggesting GV possibly affect IMT through PI3K or AMPK pathway.

Verona Diabetes Complicated Study [27] previously postulated that HOMA-IR was also an significant predictor of cardiovascular disease in type 2 diabetes. ApoE2/2 mice without insulin resistance, which had a single allele of the insulin receptor deleted, will not enhance the severity of atherosclerosis [28]. It has long been known that the insulin resistance in type 2 diabetes is caused by decrease in receptor concentration and kinase activity, the concentration and phosphorylation of insulin receptor substrate-1/−2, PI3K activity, and glucose transporter translocation [29]. Thus insulin resistant plays a significant role on mac-complications, especially atherosclerosis. Now that GV could affect insulin resistant, so the improvement of GV might have beneficial effects not only on glucose control but also on CVD in type 2 diabetes.

Evidence continues to point to PI3K, which is only common between insulin resistant and IMT, and AKT is activated downstream of PI3K. It is also consistent that people with impaired glucose tolerance show a higher IMT [22]. As our knowledge, PI3K/Akt mediates recruitment of glucose transporter GLUT4 and also enhances glucose oxidation, and it can effect endothelial function and inhibit cell apoptosis of myocardial cells. So according to our meta-analysis, we speculated that the one pathogenesis of GV is probably to affect PI3K/AKT single pathway, then aggravated glucose tolerance and increased IMT levels, further leaded to CVD events.

Although no statistical significance between lipid metabolism and GV in this meta-analysis, change of TG was effected by GV. Because the limited study number, short trial duration, and inconsistent of GV index may contribute non-statistical. As my knowledge, TG can be possibly maintained to prevent insulin resistance. Hypertrophy of adipocytes on overloading TG significantly increases inflammatory status, especially tumour necrosis factor-α (TNF-α) [30]. The reason is possibly that TG is the main maker to affect insulin resistance in the lipid metabolism. So the key question is what are the mechanisms on the reduced TG in type 2 diabetes? Inflammatory factor, especially TNF-α, may be effected by GV, because of limitation of studies, we failed to explore the association between them. More theoretical work is needed to better understand the mechanism of GV, how it may be related to outcomes of interest and how to effectively change TG and inflammatory factor.

This meta-analysis has some limitations that should be considered. First, some of the studies had small sample size, especially meta-analysis of IMT levels. So caution is needed in the interpretation of the result from the meta-analysis, since the meta-analysis may have been underpowered. Second, in this meta-analysis, some studies were heterogeneous in terms of demographic characteristics and clinical features. This heterogeneity, as well as confounding factors such as different treatment, different measuring methods and limited clinical information, could affect the results. Nevertheless, the meta-analysis also has advantage. A strength of this study is to include studies published in English and Chinese languages, and no publication restrictions, all available data were included, thereby increasing the power of the study.

## Conclusion

The healthy people maintain a balance in glucose uptake and production, and the GV could break the balance. And this meta-analysis show that minimizing GV is effective in improving the insulin resistance and IMT that are associated directly with cardiovascular disease. In other words, this analysis indicates that HOMA-IR and IMT possibly play an important role in glucose variability pathogenesis. Further studies are needed to determine how GV directly contribute to the pathogenesis of CVD in detail.

## Additional file

Additional file 1:
**Figure S1.** Effect of GV on BMI in type 2 diabetes. **Figure S2.** Effect of GV on WC in type 2 diabetes. **Figure S3.** Effect of GV on HDL in type 2 diabetes. **Figure S4.** Effect of GV on LDL in type 2 diabetes. **Figure S5.** Effect of GV on TC in type 2 diabetes. **Figure S6.** Effect of GV on Hs-CRP in type 2 diabetes. **Figure S7.** Effect of GV on HOMA-βin type 2 diabetes (DOCX 2673 kb)

## Abbreviations

AMPK: AMP-activated protein kinase
BMI: Body Mass Index
CVD: Cardiovascular disease
GV: Glucose variability
HbA1c: Glycosylated hemoglobin
HDL-C: HDL cholesterol
HGVG: High glucose variability group
HOMA: Homeostasis model assessment
Hs-CRP: High-sensitivity C-reactive protein
IMT: Carotid intima-media thickness
LDL-C: LDL cholesterol
LGVG: Low glucose variability group
MD: Mean difference
PI3K: Phosphoinositide 3-kinase
SMD: Standardized mean difference
TC: Total cholesterol
TG: Triglyceride
TNF-α: Tumour-necrosis factor-α
WC: Waist circumference
