# Supplementary material for: Glucose variability for cardiovascular risk factors in type 2 diabetes: a meta-analysis
Source: J Diabetes Metab Disord. 2017 Nov 14;16:45. doi: 10.1186/s40200-017-0323-5 (PMC5686902; doi:10.1186/s40200-017-0323-5)
Supplement: Additional file 1: — Figure S1. Effect of GV on BMI in type 2 diabetes. Figure S2. Effect of GV on WC in type 2 diabetes. Figure S3. Effect of GV on HDL in type 2 diabetes. Figure S4. Effect of GV on LDL in type 2 diabetes. Figure S5. Effect of GV on TC in type 2 diabetes. Figure S6. Effect of GV on Hs-CRP in type 2 diabetes. Figure S7. Effect of GV on HOMA-βin type 2 diabetes (DOCX 2673 kb) [file 40200_2017_323_MOESM1_ESM.docx]

**

**



Figure S1 Effect of GV on BMI in type 2 diabetes Figure S2 Effect of GV on WC in type 2 diabetes







Figure S3 Effect of GV on HDL in type 2 diabetes Figure S4 Effect of GV on LDL in type 2 diabetes





Figure S5 Effect of GV on TC in type 2 diabetes





Figure S6 Effect of GV on Hs-CRP in type 2 diabetes





Figure S7 Effect of GV on HOMA-βin type 2 diabetes
